# In This Issue: Volume 116, Issue 6, June 2025

**DOI:** 10.1111/cas.70104

**Published:** 2025-06-01

## Permeable Lung Vasculature Creates Chemoresistant Endothelial Niche by Producing SERPINE1 at Breast Cancer Metastatic Sites

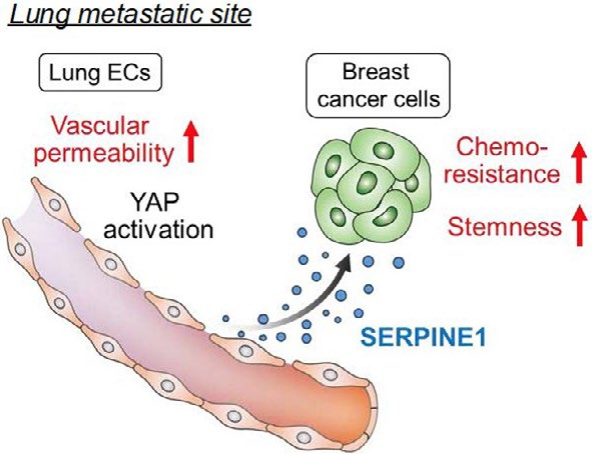

Triple‐negative breast cancer is an aggressive form of breast cancer that often spreads to other organs, especially the lungs. Cancer cells that have spread to other organs (called metastasis) have been found to overcome chemotherapy, making it hard to treat. Hongu et al. have explored how these metastatic cells escape treatment, using breast cancer cells that have spread to the lungs in mice. They found that the main problem was not just the cancer cells, but the cells lining the blood vessels (endothelial cells).

When cancer develops, and the blood vessels become leakier, the endothelial cells lining these blood vessels have reduced contact with one another. This, in turn, activates a signaling cascade called the YAP‐TEAD pathway, which leads to an increase in secretion of a protein called SERPINE1 from the endothelial cells. The researchers found that when SERPINE1 levels are high, patients are less likely to respond to chemotherapy. They observed that the presence of this protein around the blood vessels helped the metastatic cells escape chemotherapy drugs, a phenomenon called chemoresistance. This interaction between the cancer cells and the endothelial cells creates a protective environment for the metastatic cells, around these blood vessels, promoting stemness in addition to chemoresistance.

The researchers further probed how this chemoresistance can be overcome. They showed that blocking SERPINE1 using a drug (tiplaxtinin) made chemotherapy more effective in mice with lung metastases. This combination treatment significantly reduced the spread of breast cancer in the lungs compared to chemotherapy alone. Addition of such drugs can block SERPINE1 or make the leaky blood vessels healthy and less leaky again, along with the chemotherapy. This study has shed new light on the signaling between various cells in cancer metastasis and the advantages of fixing the cancer blood vessels for effective killing of metastatic cells.

https://onlinelibrary.wiley.com/doi/full/10.1111/cas.70050

## Claudin‐11 Enhances Invasive and Metastatic Abilities of Small‐Cell Lung Cancer Through MT1‐MMP Activation

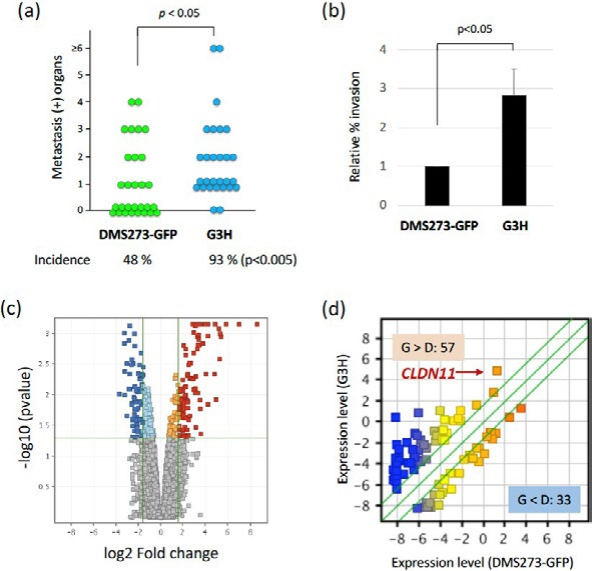

Small cell lung cancer (SCLC) is an aggressive malignancy that rapidly spreads, or metastasizes, to other tissues and organs. Only 5%–10% of patients survive 5 years after an SCLC diagnosis. Understanding the mechanisms of SCLC metastasis is crucial for developing new treatments. Orthotopic transplantation—transplanting tissues and organs into their normal anatomical location—of human SCLC cells into the lungs of mice is a valuable model for studying how SCLC spread to distant tissues and organs. But what are the molecular factors driving this metastatic behavior?

A transmembrane protein called Claudin‐11, which forms tight junctions between adjacent cells to maintain tissue integrity, may play a key role. When claudin‐11 expression is suppressed, cell‐to‐cell adhesion is disrupted in many tissues, allowing cells to become mobile—a precursor to metastasis. This phenomenon has been observed in gastric, nasopharyngeal, and squamous cell cancers. However, its role in SCLC remains poorly understood.

In this study, Sakamoto et al. investigated the role of claudin‐11 in the progression and metastasis of SCLC. The researchers employed an orthotopic transplantation model; transplanting DMS273, a human SCLC cell line, into mice. Microarray analysis revealed that claudin‐11 overexpressed in GH3 cells, a highly metastatic subline of DMS273 cells, with the protein was predominantly localized to the cell membrane. Furthermore, claudin‐11 expression was observed in multiple SCLC cell lines. Gene expression data of various SCLC patients revealed that patients with higher levels of claudin‐11 correlated with poor prognoses. Claudin‐11 overexpressing cells in orthotopic mice showed increased metastasis, and more organs tested positive for metastasis. These findings confirmed that claudin‐11 promotes SCLC metastasis. But what is the underlying mechanism?

Digging deeper, the researchers discovered that claudin‐11 enhances the activity of membrane‐type 1 matrix metalloproteinase (MT1‐MMP)—an enzyme that breaks down extracellular matrix and facilitates cancer invasion. Silencing the *MT1‐MMP* gene suppressed the invasive and metastatic ability of G3H cells. Moreover, *MT1‐MMP* silencing in DMS273 cells reversed the increased metastatic potential induced by claudin‐11 overexpression.

These findings reveal a new mechanism: claudin‐11 promotes SCLC metastasis by enhancing MT1‐MMP activity, highlighting the claudin‐11/MT1‐MMP axis may be a promising target for future therapies aimed at halting the spread of SCLC.

https://onlinelibrary.wiley.com/doi/full/10.1111/cas.70038

## Differentiation of Cytotoxic CD8^+^ T Cell Subsets Under Tumor Progression: Can CD69 Be a New Therapeutic Target?

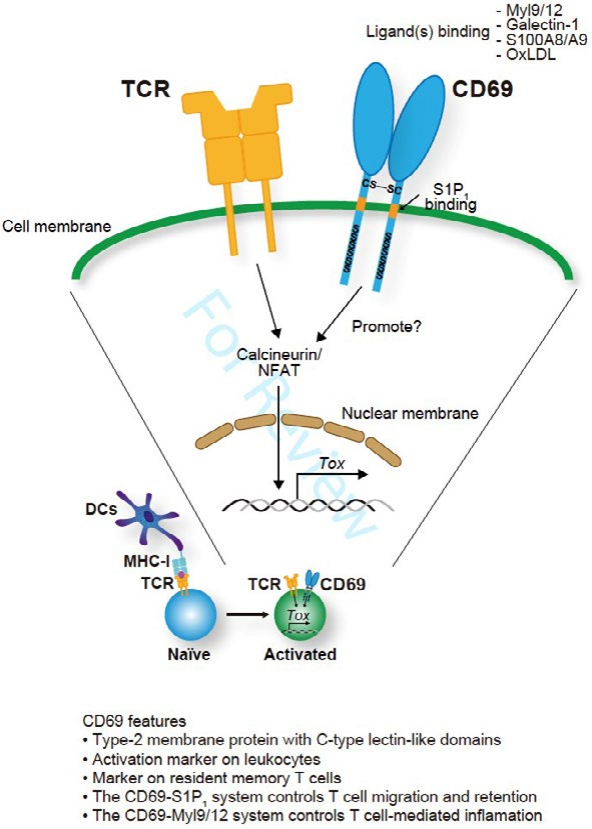

Cancer immunotherapy is a new and promising treatment strategy that helps a patient's own immune system fight cancer. While many different immune cells are involved in the anti‐tumor immune response, one of the most important among them are cells called CD8^+^ T cells. CD8^+^ T cells can be involved in both acute (or short‐term) and chronic (or long‐term) immune responses, but it is the latter that is very important for anti‐tumor immunity. Understanding the chronic CD8^+^ T cell response is therefore key to improving a patient's anti‐tumor immune response.

In this review article, Koyama‐Nasu et al. suggest that a protein called CD69 may be the key to understanding and enhancing anti‐tumor immunity. Previous studies have shown that blocking CD69 function led to enhanced anti‐tumor immunity and reduced tumor growth in mice. However, the question that arises is: how exactly does CD69 influence the anti‐tumor immune response?

To answer this, the authors explain that there are two kinds of CD8^+^ T cells involved in the chronic immune response—‘stem‐like’ CD8^+^ T cells, which then differentiate (or develop) into ‘terminally differentiated’ CD8^+^ T cells that have the ability to kill cancer cells. They hypothesize that CD69 may play a crucial role in controlling this process, potentially through effects on another protein called TOX. More specifically, they propose that blocking CD69 function reduces TOX levels in tumor‐specific CD8^+^ T cells. This in turn promotes the differentiation of ‘stem‐like’ CD8^+^ T cells into cancer‐killing ‘terminally differentiated’ cells, thus enhancing anti‐tumor immunity.

How can this information improve our current approaches to immunotherapy? The authors point out that combining anti‐CD69 therapy (which blocks CD69 function) with anti‐PD1 therapy (a type of anticancer drug) improved anti‐tumor immunity in mice. In addition, mice which lack CD69 in their body are healthy with no obvious defects, suggesting that anti‐CD69 therapy may have a favorable safety profile.

Overall, these findings show that targeting CD69 could be an effective strategy to control the CD8^+^ T‐cell chronic immune response and thus enhance anti‐tumor immunity.

https://onlinelibrary.wiley.com/doi/full/10.1111/cas.70055

